# Immediate Implant and Customized Healing Abutment for a Periodontally Compromised Socket: 1-Year Follow-Up Retrospective Evaluation

**DOI:** 10.3390/jcm12082783

**Published:** 2023-04-09

**Authors:** Giovanni-Battista Menchini-Fabris, Saverio Cosola, Paolo Toti, Myoung Hwan Hwang, Roberto Crespi, Ugo Covani

**Affiliations:** 1 Department of Stomatology, Tuscan Stomatologic Institute, Foundation for Dental Clinic, Research and Continuing Education, 55041 Camaiore, Italy; 2 San Rossore Dental Unit, San Rossore Private Hospital, 56122 Pisa, Italy; 3 Department of Dentistry, Unicamillus International Medical University, 00100 Rome, Italy; 4 New Smiles Dental Implant Center Galleria, 2930 Chimney Rock Rd, Houston, TX 77057, USA

**Keywords:** dental implant-abutment design, dental implant loading, immediate, dental prosthesis, tooth extraction, wound healing, dental implants, X-ray computed tomography

## Abstract

Immediate dental implant placement with or without immediate loading is reported in daily dentistry and implantology, but these procedures are not common in the case of periradicular and periapical lesions around the tooth needed to be replaced. In the following retrospective evaluation, 10 cases with a 1-year follow-up were selected to propose the technique of an immediate provisional non-loading prosthesis being delivered on the same day of the post-extraction implant placement in multiradicular teeth affected by chronic periradicular and periapical lesions. Post-extractive sockets underwent immediate dental implant placement by filling the empty space with sterile, re-absorbable gelatin sponges. The widths of the alveolar ridge were measured on three-dimensional radiographs before and after the operation, 4 and 12 months later. Non-parametric statistics were performed to compare the outcomes over time with a level of significance of 0.05. Comparing the preoperative cross-sectional images of cone beam computerized tomography (CBCT) scans to the postoperative ones, it was noted that changes in the crestal ridge width, ΔCW, (compared to baseline) were negligible and not clinically appreciable. However, while ΔCW at 4 months appeared to be negative (−0.17 ± 045 mm), crestal width at 12 months was at the same level as the baseline (ΔCW = 0.02 ± 0.48 mm), with a significant difference between 4 and 12 months (*p*-value = 0.0494). Immediate implant placement with an immediate non-loading provisional customized healing abutment of polyether-ether-ketone placed into the post-extractive sockets with asymptomatic and large chronic periapical and periradicular lesions could represent a further treatment strategy for patients’ rehabilitation and soft tissue preservation to replace a hopeless tooth.

## 1. Introduction

Nowadays, immediate dental implant placement with or without immediate loading is a relatively common procedure in implant dentistry if there are indications and conditions of the bone, patients, and surgeons [1].

More often than immediate loading, immediate implant placement after the extraction is considered the gold standard approach to reducing early bone resorption, especially in the aesthetic areas. Implant placement into fresh extraction sockets and immediate restoration have become widely accepted precisely because this procedure could allow clinicians to preserve the interdental papillae of the frontal areas so that soft tissue around the crown and marginal bone [2]. For a predictable immediate placement of dental implants into maxillary frontal sites, certain surgical protocols have to be followed, such as provisional restoration of the failing tooth, atraumatic tooth extraction, implant placement towards the palatal aspect of the socket, customized abutment insertion, and adequate management of the soft tissue [2,3].

Some studies have suggested sealing the fresh sockets after implant placement with a customized healing abutment manufactured from various materials: polyetheretherketone, polymethyl methacrylate, zirconia, resin composite, and titanium [4,5]. Closing the gap between the fixture and the external part of the post-extractive sockets with customized healing abutments gave results at least as predictable as those of sockets sealed with bone substitute materials and collagen [4].

Some authors used Computer-Aided Design (CAD) and Computer-Aided Manufacturing (CAM) technology to fabricate custom healing abutments before or just after the surgery in order to immediately load the fresh socket implant with a substantially lower number of steps for prosthetic finalization [6,7]. Moreover, some additional requests in the immediate implant positioning are a sufficient bone volume, apically and palatally, a fully intact facial bone wall with a thick wall phenotype (≥1 mm), a thick gingival biotype, and an absence of acute infection in the extraction site [8]. On the abovementioned topic, this type of procedure was generally contraindicated when the site was not completely infection-free or showed a generally damaged extraction socket [9,10].

Moreover, the procedure required an accurate debridement of the diseased post-extractive socket without reducing the primary stability nor affecting the anatomic and final esthetic results [11,12].

The specific cases of vestibular defects could be treated during the immediate implant placement with bone substitutes and a resorbable collagen barrier to improve the esthetic outcomes [13].

Computer-guided surgery with mucosal-supported silicone templates is reported to be a predictable procedure for implant placement and could reduce errors in the actual implant positioning after planning [14].

Even if clinicians had nowadays the option to choose among several treatment approaches to perform an immediate implant placement, the right strategies are those that meet the following conditions: infection-free or infection management, flapless procedure, intact facial bony plate, and thick gingival biotype [15].

The next step of patients’ needs pushes clinicians to give patients the possibility of having an immediate provisional non-loading prosthesis delivered on the same day of extraction and implant placement, even if the site showed periradicular and periapical lesions.

The novelty lies in the fact that, while immediate dental implant placement with or without immediate loading is reported in daily dentistry and implantology, these procedures are not common in the case of chronic periradicular and periapical lesions around the tooth needed to be replaced. This is the idea behind the following retrospective evaluation with regard to the immediate implants placed into infected sockets with periradicular chronic lesions around the roots of teeth and Polyether-ether-ketone (PEEK) abutment as a customized healing screw.

The aim of the study was to test the effectiveness and follow-up one-year clinical and radiological outcomes for an immediate implant technique with immediate non-loading through a provisional, customized healing abutment of polyether-ether-ketone. Dental implants were placed into post-extractive sockets suffering asymptomatic but large chronic periapical and periodontal lesions.

## 2. Materials and Methods

A group of patients who underwent post-extractive implant placement with one-stage surgery screwing a customized healing abutment of PEEK was enrolled from the clinic database of the Dental Unit of the San Rossore Private Hospital, Pisa, Italy. As a prerequisite of the patients’ selection, it was mandatory to have a periodontally compromised socket of the tooth needing extraction and a pre-operative and post-operative cone-beam computed tomography (CBCT) to assess the intraosseous level of infection, to better localize the position of the infected site, and to check short-term tissue healing.

Whole treatments were accepted by the patient who signed an informed consent form for medical treatment and usage of his personal information according to the Declaration of Helsinki of 2008, updated in 2013, to analyze the radiographs and clinical pictures retrospectively.

This clinical evaluation was approved by the Local Ethical Committee for retrospective data analysis at the University of Pisa (2626-2008 PROT n° 58183).

### 2.1. Inclusion/Exclusion Criteria

Patients were included if the following conditions were met:

- 18 years old or older;
- The consensus at the time the data processing had begun;
- Molar or premolar maxillary sites underwent tooth extraction and immediate dental implant placement into a severely periodontally compromised socket with light buccal and palatal bone walls but without defects.
- Immediate provisionalization of implant-supported customized healing abutment without neither any occlusal contact nor lateral excursion;
- At least 1-year clinical follow-up after loading;
- Cone Beam Computed Tomography (CBCT) scans were obtained from routine preoperative radiographs (before tooth extraction) and at 4 and 12 months after surgery.

Patients were excluded if the medical records reported:

- Any augmentation procedure;
- Any smoking habits (>cigarettes a day), alcohol/drug abuse, or oral parafunctional habit;
- Whatever kind of file.dicom corruption.

### 2.2. Surgical Procedures

Immediately before the surgery, all patients used a mouth rinse of chlorhexidine digluconate solution 0.2% for 1 min and 2 compresses of antibiotics (1 compress: 875 mg of amoxicillin with 125 mg of clavulanic acid) 1 h prior to surgery; then, after the surgery, they continued the antibiotic treatment (1 compress every 12 h) for 5 days more. After the first day of the surgery, for one week, patients were asked to use the mouthwash with chlorhexidine digluconate 0.2% every night after normal oral hygiene procedures for local decontamination purposes.

Loco-regional anesthesia using lidocaine with epinephrine (1:50,000) was performed.

Before extraction, the residual crown underwent sectioning in order to produce 2–4 single-root pieces so as to ensure the least traumatic, minimally invasive procedure possible without any flap elevation.

**Figure 1:**
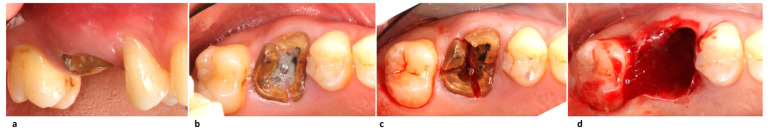
Less traumatic removal of the residual roots of the maxillary molar tooth. Preoperative area lateral (**a**) and occlusal views (**b**); residual roots separated from each other with one root per broken tooth segment (**c**); post-extractive site (**d**).

Luxation of each fragment was performed by a magnetoelectric device (Magnetic Mallet, www.osseotouch.com, accessed on 13 December 2022, Turbigo, Milano, Italy) used to sever the remaining cervical gingival attachment fibers with shock waves of 130 daN through a MM-F-EXTR-2D blade applied on the tip of the Mallet. Each shock wave allowed an easy and atraumatic blade penetration of about 2 mm at a time by mechanically separating the root fragment from the rest of the tissues at the mesial and distal aspects in order to preserve an intact bony septum at the level of furcation point. Then each fragment was extracted with extraction forceps for residual roots, proceeding with a rotational force in a coronal direction (Figure 1).

**Figure 2:**
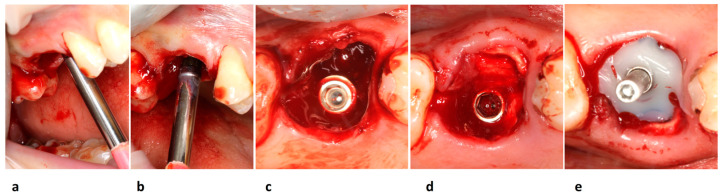
Dental implant placement steps. Implant bed preparation and control of implant direction in the mesial (**a**), and lateral view (**b**); occlusal view of placed dental implant (**c**); view of re-absorbable gelatin sponge around implant within the bone defect (**d**); screwed PEEK core healing abutment covered with moldable composite resin (**e**).

Before dental implant placement, the post-extraction socket underwent soft debridement with a Hemingway alveolar spoon with a double-sided bend. The apical implant bed was created in the area of the interradicular septum, and after verifying the proper implant direction, titanium plasma spray implants with an external hexagonal connection, a body with a progressive thread design, a sandblasted and acid-etched surface, and pure-grade 4 titanium (Pro-Link Out-Link, Sweden & Martina, Due Carrare, Padua, Italy), between 8.5 and 11.5 mm in length and 4.1–5 mm in diameter, were positioned (Figure 2) using an insertion torque of ≥30 Ncm [16]. Considering the pre-operative clinical situation of the tooth site, there is no reverse torque measurement after implant placement.

The defect between the implant and the inner walls of the post-extraction socket was filled with a sterile, re-absorbable gelatin sponge (Cutanplast^®^ Dental, DispotechS.r.l., Gordona, SO, Italy). No suture was required because the collagen and blood clots inside the socket were stabilized by the provisional customized healing abutment (Figure 2), and the socket’s soft tissues were sustained.

**Figure 3:**
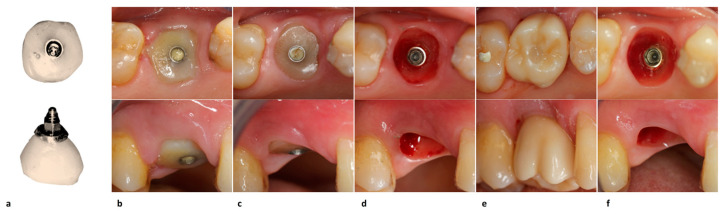
Prosthetic steps with occlusal (upper) and lateral views (lower). Semi-finished peek-core customized healing abutment (**a**); healing of mucosa around the PEEK core abutment covered with composite resin 1 week after surgery (**b**); implant site with (**c**) and without (**d**) the finished customized healing abutment 1 month after surgery; restored implant with (**e**) and without (**f**) single crown fixed prosthesis 3 months after surgery.

The core of the surgical abutment was fabricated by mimicking the emergence profile of the tooth being replaced via a virtual CAD/CAM procedure and subtractive milling in polyether ether ketone material (PEEK, BioHPP, Bredent Medical GmbH & Co.KG, Weissenhorner, Germany), as seen in Figure 3. The polyether-ether-ketone (PEEK) was chosen because of its biocompatibility and the possibility of creating a personalized healing abutment in an easy and fast way through a digital milling machine. When necessary, a moldable composite resin was used to cover the customized peek-core to better adapt the shape of the healing abutment to the post-extractive socket. Then, a modified pre-made straight post embedded in the peek core was engaged to the implant by using a retentive titanium fixation screw.

### 2.3. Postoperative Prosthetic Management

Four weeks after surgery, soft tissues around the healing abutment appeared to be healed but were certainly not yet aesthetically perfect. During the next two months, the provisional crown was then modified to guide them to a perfect appearance or, at least, to what seemed proper for the clinician. Finally, about 90 days after surgery, the implant was loaded with a definitive screwed metal-ceramic crown (Figure 3).

To monitor the bone status around dental implants, radiographs were investigated. It was assumed that an implant was osseointegrated if the surrounding bone showed no radiolucent area and the patient’s case sheet did not report signs or symptoms of implant failure such as the presence of mobility, suppurative mucosa, or associated pain, either spontaneous or due to the application of lateral force.

### 2.4. Variables and Outcomes

Primary predictors: There were three-time points in the test period. Pre-operative time point was the baseline; then, variables were generally measured 4 and 12 months after surgery.

Covariates: They were the variables for sample description: age, gender, and tooth position.

Variables: They described characteristic clinical and radiological features of the sample and they were as follows:

- A computerized tomography scan was acquired using a dedicated device (Gendex GXCB-500; Gendex Dental Systems, Hatfield, PA, USA). A matrix elaborator (MatLab 7.11, The MathWorks, Natick, MA, USA) superimposed files from each patient with the following settings: isotropic voxel 300 µm, Field of View 84 mm, 16 bits. Then, a dentascan software (SimPlant 12.02, Materialise Dental Italia, Rome, Italy) allowed a single-blind examiner (TP) to perform all the measurements [17,18]. Alveolar widths (AWs at 4, and 12 months) were measured on the superimposed cross-sectional image (with the implant as the reference object). The AW was the length of the segment perpendicular to the implant direction and passed 1 mm apical from the most prominent buccal point to the most coronal point of the palatal cortex. The change in alveolar width (ΔAW) was described by Equation (1).
  (1) $$∆∆AW={AW}_{1yr}-{AW}_{bsl}$$
- The Papilla Index (PI) of Jemt registered the position of papillae between the adjacent teeth and the surgical area according to the following criteria: 0 = no papilla; 1 = less than 50% of the papilla height; 2 = at least 50% of the papilla height but not all the interproximal space; 3 = papilla filling the interproximal space and with a favorable gingival contour; 4 = hyperplastic papilla and an unfavorable gingival contour [19]. For this purpose, for each papilla, the reference was the line through the zenith of the implant restoration to that of the adjacent tooth.
- Peri-implant Probing Depths (PDs) were assessed with a periodontal probe (Hu-Friedy PGF-GFS, Hu-Friedy, Chicago, IL, USA) and reported to the nearest millimeter [20].
- Keratinized Mucosa (KM) width was measured at the mid-buccal point by a periodontal probe as the distance between the mucogingival junction and the free gingival margin. Changes in Keratinized Mucosa Width, ∆KM, were measured by Equation (2):
  (2) $$∆KM={KM}_{postoperative}-{KM}_{preoperative}$$

### 2.5. Statistical Analysis of Outcomes

Descriptive statistics for patients’ characteristics and treatment outcomes were conducted using Microsoft Excel-Office 365 (Statistics Toolbox, MatLab 7.11; The MathWorks Natick, Boston, MA, USA) to summarize and analyze the differences between time points according to complications, the clinical intervention, and the type of extraction. The Gaussian distribution was not confirmed by the Shapiro–Wilk analysis. Measurements repeated in time (preoperative and postoperative) were matched to the data; each pair-wise comparison was performed by the Wilcoxon signed-rank test. The variables are described as mean ± standard deviation (rounded to the nearest decimal). The level of significance was set at 0.05.

## 3. Results

Here, 10 patients, aged between 35 and 61 years, were included in the evaluation; 7 were female and 3 were male. The demographic data are described in Table 1. Out of 10, 6 cases were molars and the remaining 4 were premolars. The survival rate was 100%, and no infections, early failures, mucositis, or peri-implantitis had been reported in the observational period. The clinical and radiographic outcomes are shown in Table 2.

**Figure 4:**
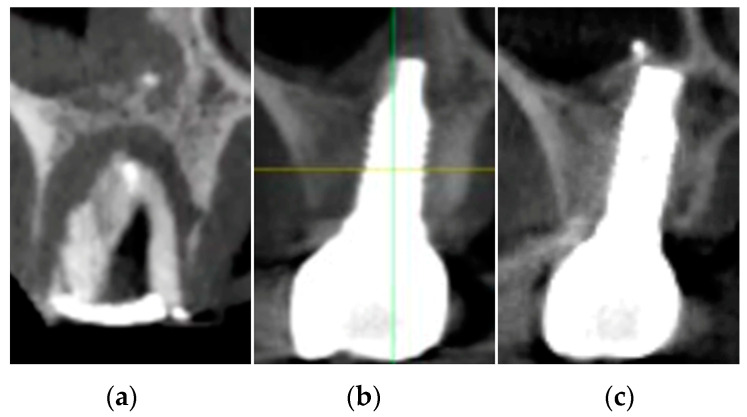
Cross-sectional images: preoperative (**a**); post-surgical at 4 months (**b**); and at 1 year (**c**).

In the case presented with pictures and radiographs, the molar underwent endodontic therapy approximately two years before, but despite this, the clinical examination evidenced a third degree of mobility with the presence of intact buccal and palatal plates at a radiological three-dimensional investigation. Moreover, the presence of a very deep pocket all around the tooth led to the initial diagnosis of severe intrabony periodontal destruction (Figure 4).

These cases confirmed the clinical advantages of performing a personalized non-loading provisional healing abutment immediately after implant placement because, while comparing the preoperative cross-sectional images of CBCT to the postoperative ones (at 4 and 12 months), it was noted that at 4 months the change in crestal width appeared slightly reduced (−0.17 ± 0.45 mm) in length but reaching the statistical significance (*p*-value = 0.0494), and the site at 12 months healed with a real “restitutio ad integrum” (Figure 4); in fact, the crestal width at 12 months (8.96 ± 1.33 mm) appeared to be very similar to the baseline value, 8.94 ± 1.32 mm (without any significant difference).

Regarding clinical results, no significant differences are registered for probing depth and Jemt Papilla index (values are reported in Table 2). However, keratinized bandwidth appears to increase from 4 months (2.6 mm) to a 1-year survey (3.0 mm), with a *p*-value = 0.0367.

## 4. Discussion

Immediate implant placement was meant to prevent or mitigate alveolar bone resorption at the site of tooth removal, as suggested by many different studies [21,22].

However, preservation of the alveolar ridges after tooth extractions was, in fact, never complete, but still, augmentation procedures contextual to the immediate implant positioning might just balance tissue gains and losses, according to the literature [23].

There were very limited evidence and outcomes of immediate implants positioned in chronic periapical infected areas without tissue regeneration, and some authors reported even that the early implant placement (after 8–12 weeks from the extraction) appeared more effective in preserving peri-implant marginal bone level than immediate procedures [24,25,26].

The immediate positioning of dental implants into chronically infected periapical and periodontal sites was evaluated by some authors in a few recent reviews as being of low or moderate treatment quality, mainly due to several risk factors for early failure due to incomplete bone healing, fibro-integration, or marginal bone loss. However, according to the authors themselves, given the presence of several uncontrolled confounding factors, the results regarding survival rates and patients’ prognoses appear to be very contradictory [27,28] because the timing of implant loading has not been considered. On the contrary, the present retrospective evaluation showed that a severe chronic periradicular and periapical intrabony defect of a multi-rooted tooth could be treated using a fresh socket implant and immediate provisionalization with a customized healing abutment produced using a PEEK-core and an external casing in composite resin.

In the literature, no studies have yet examined the impact of immediate provisional with customized healing abutment on alveolar width at the implant crestal level. However, some studies describing the buccal width of keratinized mucosa at the site of the implantation (with a range from 3.2 mm to 3.64 mm) reported results very similar to that of the present study (3 mm after 1 year of the survey) [29,30,31]. Moreover, frequently the studies described data regarding frontal sites.

In the present clinical evaluation, the computed tomography preoperative cross-sectional image revealed the signs of severe bone demineralization, as reflected by extensive and chronic periapical periodontitis that occurred all around the tooth roots. Some authors suggested that similar radiotranslucent lesions necessarily required teeth extraction and, generally, antibiotic administration, and also meticulous alveolar debridement, before dental implant placement [32].

In this view, the present retrospective evaluation reported the use of a protocol with an immediate non-loading provisional customized prosthesis for a single implant placed into a posteriorly infected socket for the purpose of replacing a tooth suffering from an extensive and chronic periapical lesion. The present protocol agreed with the findings of Sculean and co-workers about the self-regenerative potential of intraoral bone defects [33] with a substantial contribution to maintaining the dimensional width of the treated posterior alveolar areas. In fact, authors suggested that several high self-regenerative capacities of a bone defect appeared only if there was a set of boundary conditions and limits such as the presence of intact bony walls, healing enclosed space, enough space to heal, and mechanical stability for wound healing [33].

So, the present strategy was based on completely covering all exposed healing soft and hard tissues of the post-extraction socket with an immediate and customized screw-retained healing abutment without any kind of contact, i.e., neither lateral nor occlusal. This healing abutment protects the bony walls, giving space to the coagulum and healing process, giving mechanical stability to the periosteum otherwise contracted by myofibroblast for wound healing, and without any risk of overloading the implant before the biological stability has overcome the primary stability [34].

No regenerative biomaterials (bone substitute materials) were used in association with the implant tent effect, apart from the use of a highly absorbable collagen sponge within the socket. So, the present sealing approach seemed sufficient to almost guarantee a complete recovery, given the present result [35].

Therefore, it became fundamental for a bone defect to be self-regenerative after dental implant placement. So, a flapless extraction of the tooth was imperative to respect buccal and palatal bone walls and to protect the coagulum [36].

Given the nature of the study, it was not possible to extend the present results on the preservation of the horizontal dimension of the posterior alveolar bone to the anterior aesthetic area suffering periradicular and periapical bone lesions, although some studies have suggested that immediate healing abutment might allow for good maintenance of the alveolar width [37,38]. Moreover, the retrospective nature of the study affects the size of the sample, which appeared to be restricted due to the need for superimposing preoperative and postoperative tomography scans, and it is not designed to allow the drawing of conclusions about implant survival rate. The present proposed protocol should be evaluated by longer prospective clinical studies in different conditions.

## 5. Conclusions

A protocol that consists of fresh socket implant placement with immediate non-loading provisional customized polyether-ether-ketone/composite resin healing abutment could represent a valid treatment strategy for rehabilitation of maxillary posterior multiradicular asymptomatic infected teeth with post-extractive sites presenting large bone defects due to chronic periradicular and periapical lesions. The study showed promising results regarding the preservation of the alveolar width dimension and the presence of adequate buccal width of the keratinized mucosa. The present proposed surgical and prosthetic protocol of this retrospective evaluation should be deepened by longer prospective clinical studies with different materials of the customized abutment.

## Figures and Tables

**Table 1:** Anamnestic data of the sample, std: standard deviation.

Patients (number)	10
Gender (male/female)	3/7
Position (premolar/molar)	4/6
Age (mean ± std)	50.5 ± 8.8

**Table 2:** Complications and mean clinical and radiographic data during the follow-up period for each case.

Outcomes	Times	Measures	Time Comparison	Statistics (Paired)
Radiographical outcomes				
Crestal width (mm)	Baseline	8.94 ± 1.32	Baseline vs. 4 months	0.2638
	4 months	8.77 ± 1.22	Baseline vs. 12 months	0.9085
	1 year	8.96 ± 1.33	4 months vs. 1 year	0.0494 *
Changes in crestal width (mm)	4 months	−0.17 ± 0.45	4 months vs. 1 year	0.0494 *
	1 year	0.02 ± 0.48		
Clinical outcomes				
Complication or failure (count)	1 year	0		
Probing depth (mm)	4 months	2.58 ± 0.47	4 months vs. 1 year	0.9183
	1 year	2.55 ± 0.52		
Jemt Papilla index (rank)	4 months	1.95 ± 0.37	4 months vs. 1 year	0.1381
	1 year	1.70 ± 0.35		
Keratinized mucosa (mm)	4 months	2.60 ± 0.52	4 months vs. 1 year	0.0367 *
	1 year	3.00 ± 0.00		

* The mean values in bold are significantly different in time comparison with *p* < 0.05.

## Data Availability

Not applicable.
